# A useful intraoperative technique for transiliac-transsacral screws: a point-to-point coaxial guide apparatus

**DOI:** 10.1186/s13018-021-02239-2

**Published:** 2021-01-28

**Authors:** Ze-hang Zheng, Fei Xu, Zheng-qiang Luo, Ye Ren, Tao Fu, Han-qing Xu, Bin-bin Liu

**Affiliations:** Department of Orthopaedics Surgery, Tongji Hospital, Tongji Medical College, Huazhong University of Science and Technology, Wuhan, 430030 China

**Keywords:** Pelvic fracture, Transiliac-transsacral screw, Point-to-point, Guide apparatus, Corridor

## Abstract

**Background:**

The transiliac-transsacral screw placement is a clinical challenge for surgeons. This study explored a point-to-point coaxial guide apparatus assisting the transiliac-transsacral screw insertion and aimed to investigate the feasibility and accuracy of the guide apparatus in the treatment of posterior ring unstable pelvic fracture compared with a free-hand technique.

**Methods:**

A retrospective study was performed to evaluate patients treated with transiliac-transsacral screws assisted by the point-to-point coaxial guide apparatus or free-hand technique. The intraoperative data of operative time and radiation exposure times were recorded. Postoperative radiographs and CT scans were performed to scrutinize the accuracy of screws position. The quality of the postoperative fracture reduction was assessed according to Matta radiology criteria. The pelvic function was assessed according to the Majeed scoring criteria at 6 months postoperatively.

**Results:**

From July 2017 to December 2019, a total of 38 patients were included in this study, 20 from the point-to-point guide apparatus group and 18 from the free-hand group. There were no significant differences between the two groups in gender, age, injury causes, pelvic fracture type, screws level, and follow-up time (*P* > 0.05). The average operative time of the guide apparatus group for each screw was significantly less than that in the free-hand group (25.8 ± 4.7 min vs 40.5 ± 5.1, *P* < 0.001). The radiation exposure times were significantly lower in the guide apparatus group than that in the free-hand group (24.4 ± 6.0 vs 51.6 ± 8.4, *P* < 0.001). The intraosseous and juxtacortical rate of screw placement (100%) higher than in the free-hand group (94.4%).

**Conclusion:**

The point-to-point coaxial guide apparatus is feasible for assisting the transiliac-transsacral screw in the treatment of posterior unstable pelvic fractures. It has the advantages of simple operation, reasonable design and no need for expensive equipment, and provides an additional surgical strategy for the insertion of the transiliac-transsacral screw.

## Introduction

The posterior ring unstable pelvic fracture is a common type of pelvic fracture in clinic which carries severe injuries [1]. Percutaneous iliosacral and transiliac-transsacral screws fixation provides a less invasive approach for treatment of posterior pelvic ring injuries, which has several advantages including decreased intraoperative blood loss, and lower postoperative infection rates [2, 3]. Several studies have shown that standard iliosacral screws may not provide adequate mechanical fixation, especially the pelvis with vertical instability [4, 5]. In the case of a single screw, the transiliac-transsacral screw has a longer length than a standard iliosacral screw and provides contralateral cortical fixation, which is thought to provide improved resistance to vertical shear forces [6, 7]. Therefore, for patients with posterior ring unstable pelvic fracture, some surgeons, including us, prefer using transiliac-transsacral screws.

However, the transiliac-transsacral screw placement is a clinical challenge for surgeons. Its risk of extraosseous placement is theoretically increased and the operative time, the bleeding, infection, and anesthetic risk is potentially increased [2, 8]. In addition, the transiliac-transsacral screw’s starting point and trajectory are constrained and must be carefully planned and executed to avoid wayward screw placement [9]. Knowledge of the posterior pelvic anatomy, its variations, related imaging for sacral fixation is critical to identify the transiliac-transsacral screw’s safe zone and starting point [10–12]. As several previous studies mentioned and our experience, we believe the identification of the starting point and safe zone depends on improved anatomic understanding, imaging advances, preoperative screw corridor assessment, and the experience of surgeons.

Moreover, the other intraoperative difficulty of transiliac-transsacral screw insertion is to find suitable intraosseous trajectories to avoid neural compression or vascular injury and keep the trajectory and direction of the guidewire undeflected during the insertion process. The screws are inserted from the injured hemipelvis’ ilium, through the sacral body and bilateral ala, and exit the contralateral iliac cortical bone, which results in a small available bone volume [2]. It means that even a slight deflection of the guidewire may cause extraosseous placement. Also, soft tissue, a wrong lateral sacral fluoroscopic intraoperative image and body position of the patient may also interfere with the procedure [8]. All these make it difficult to find the trajectory and keep the direction of the guidewire safety. Therefore, we have been exploring a solution, point-to-point coaxial guide apparatus, to guide the trajectory and direction of the guidewire, which can address this difficulty in transiliac-transsacral screw placement.

The purpose of this study was to explore the feasibility of the point-to-point coaxial guide apparatus assisting the transiliac-transsacral screw insertion and to investigate whether the guide apparatus can improve the accuracy of transiliac-transsacral screws in the treatment of posterior ring unstable pelvic fracture.

## Materials and methods

### Participants

This study was designed as a retrospective study to evaluate patients treated with transiliac-transsacral screws assisted by the point-to-point coaxial guide apparatus or free-hand technique. Surgical placement of transiliac-transsacral screw was performed by the surgeon team in our department. The records of our hospital trauma database were retrospectively screened from July 2017 to December 2019 to identify all consecutive patients treated with posterior pelvic ring fractures. The clinical application of this technique and the retrospective review has been approved by the Ethics Committee of our university and conducted in accordance with the Helsinki Declaration of 1975 as revised in 2013. All patients have signed an informed consent form.

Inclusion criteria were (i) unstable posterior pelvic ring injuries and (ii) treatment with transiliac-transsacral screws with the technique or free-hand, and (iii) a minimum follow-up of 6 months. Patients were excluded if (i) preexisting altered skin condition and/or infection at the surgical side, (ii) treatment with transiliac-transsacral screws by other apparatuses.

### Outcome

Radiological assessment included pre- and postoperative radiographs (inlet, outlet, lateral, and anteroposterior) and CT scans. All pelvic injuries were categorized into the OTA/AO classification [13]. Postoperative radiographs and CT scans were performed to scrutinize the reduction and accuracy of screws position. Its position was defined as intraosseous, juxtacortical, or extraosseous previously [8, 9]. Complications such as nonunion, infection, nerve palsy, and paralysis were evaluated based on follow-up radiographs and retrospective chart review of the patients’ medical records.

The intraoperative data of operative time and radiation exposure times were recorded. In this study, the operative time is from the incision to the safe placement of screw. The radiation exposure times refer to the number of C-arm fluoroscopy exposures to confirm the safety of the screw placement intraoperatively.

The quality of the postoperative fracture reduction was assessed according to Matta radiology criteria: excellent (≤ 4 mm), good (5–10 mm), fair (11–20 mm), and poor (> 21 mm) [14]. The pelvic function was assessed according to the Majeed scoring criteria at 6 months postoperatively, which is based on the clinical findings including pain, work, sitting, sexual intercourse, and standing [15]. The score from 100 to 85 is classified as excellent, 84 to 70 as good, 69 to 55 as fair, and less than 55 as poor.

### Design and manufacture of the point-to-point coaxial guide apparatus

**Fig. 1:**
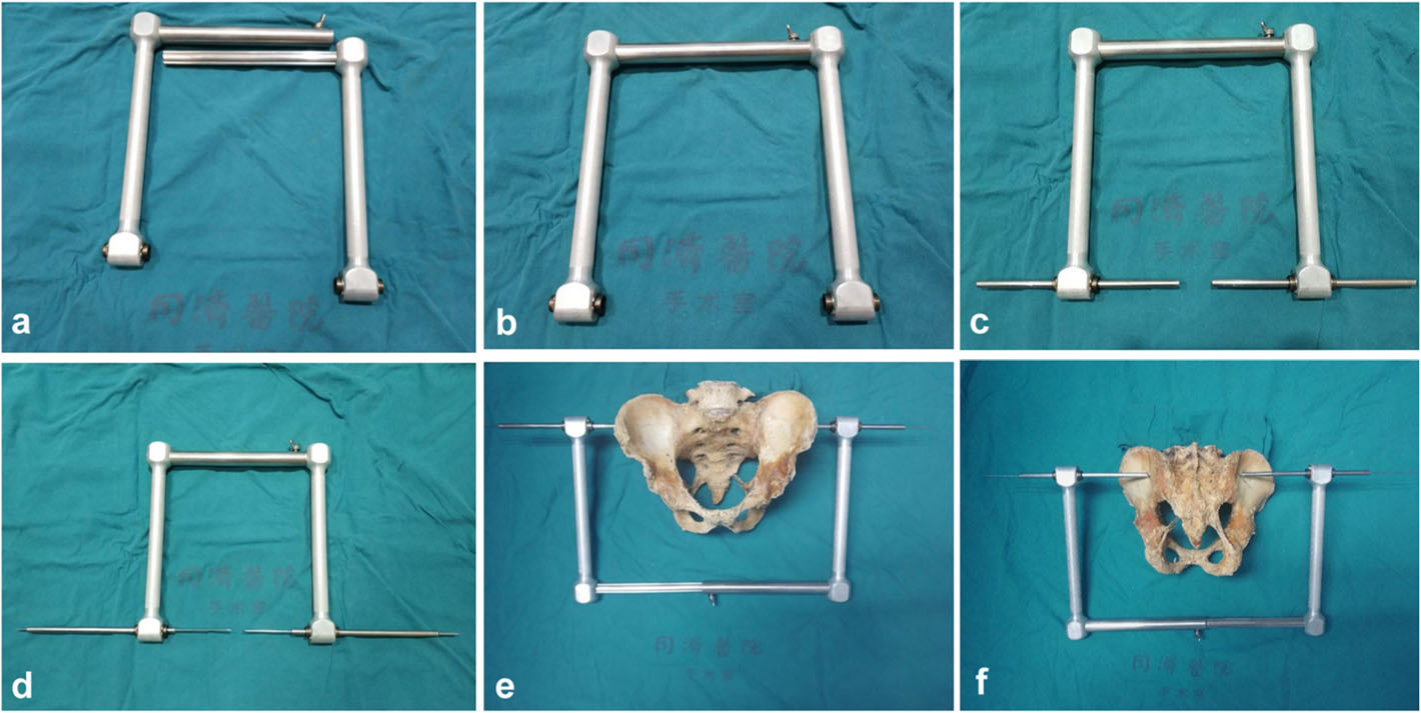
The two handles are disassembled and combined (**a**, **b**). The coaxial corridor formed at the distal end can pass two cannulas (**c**). Insert the guide wires over the cannulas (**d**). As shown at the pelvis model, the two cannulas perform a point-to-point coaxial relationship by the guide apparatus and navigate the guidewire into the corridor of the screw (**e**, **f**)

The point-to-point coaxial guide apparatus consists of two right-angled handles that can be disassembled or combined (Fig. 1a, b). When the two handles are combined, a coaxial corridor can be formed at the distal end. The coaxial corridor can pass two cannulas, which can cannulate the guidewire of the transiliac-transsacral screw (Fig. 1c). The cannulas have a serrated tip that is used to insert 2 mm into the ilium cortex. Since the starting point and the exiting point are guided by the two cannulas, the coaxial straight line formed by the cannula is the safety corridor of the screw (Fig. 1d). As shown at the cadaveric specimen of the pelvis, the two cannulas perform a point-to-point coaxial relationship by the guide apparatus and navigate the guidewire penetrating the corridor of the transiliac-transsacral screw from the starting point to the exiting point (Fig. 1e, f).

### Surgical technique

#### Point-to-point guide apparatus group

**Fig. 2:**
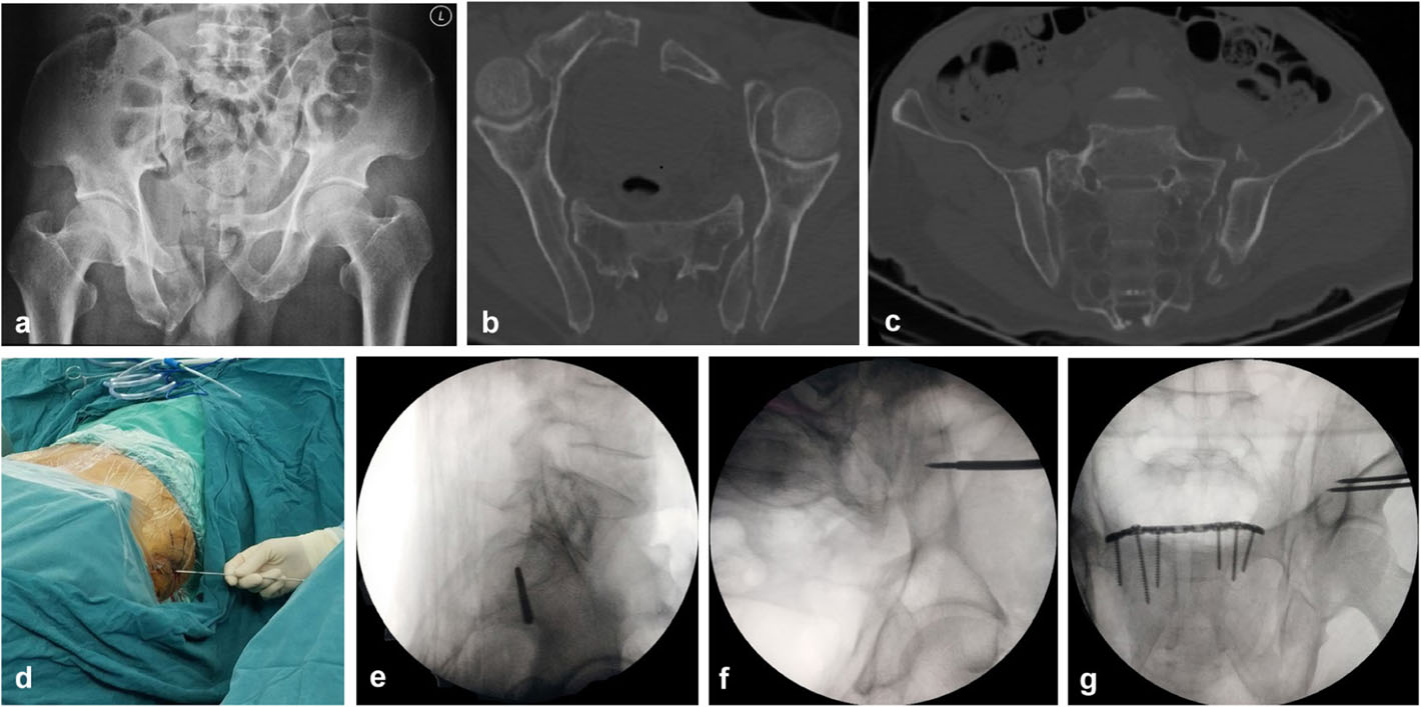
Injury anteroposterior (AP) pelvis radiograph (**a**). Reconstruction views of axial CT (**b**) and coronal CT (**c**) showed that there was a presence of dysmorphism at the upper sacral segment, and the second sacral segment existed a corridor for a transiliac-transsacral screw. The guidewire was inserted into the starting point on the fracture side (**d**) with a true lateral sacral view (**e**). Intraoperative fluoroscopic inlet (**f**) and outlet (**g**) views confirmed the position of guidewire was satisfactory

An illustrative example follows with a 56-year-old man who was involved in a car accident with a complex pelvic ring injury consisting of a complete symphysis pubis disruption, fractures of superior and inferior ramus of right pubis, vertical and rotational displacement of the left hemipelvis, and left sacroiliac joint disruption (Fig. 2a–c). At surgery, after general anesthesia, the patient was positioned supine. The reduction of the pelvic ring is achieved by axial skeletal traction and lateral compression. Symphysis pubis disruption and fractures of superior and inferior ramus of right pubis were treated with a reconstruction plate.

**Fig. 3:**
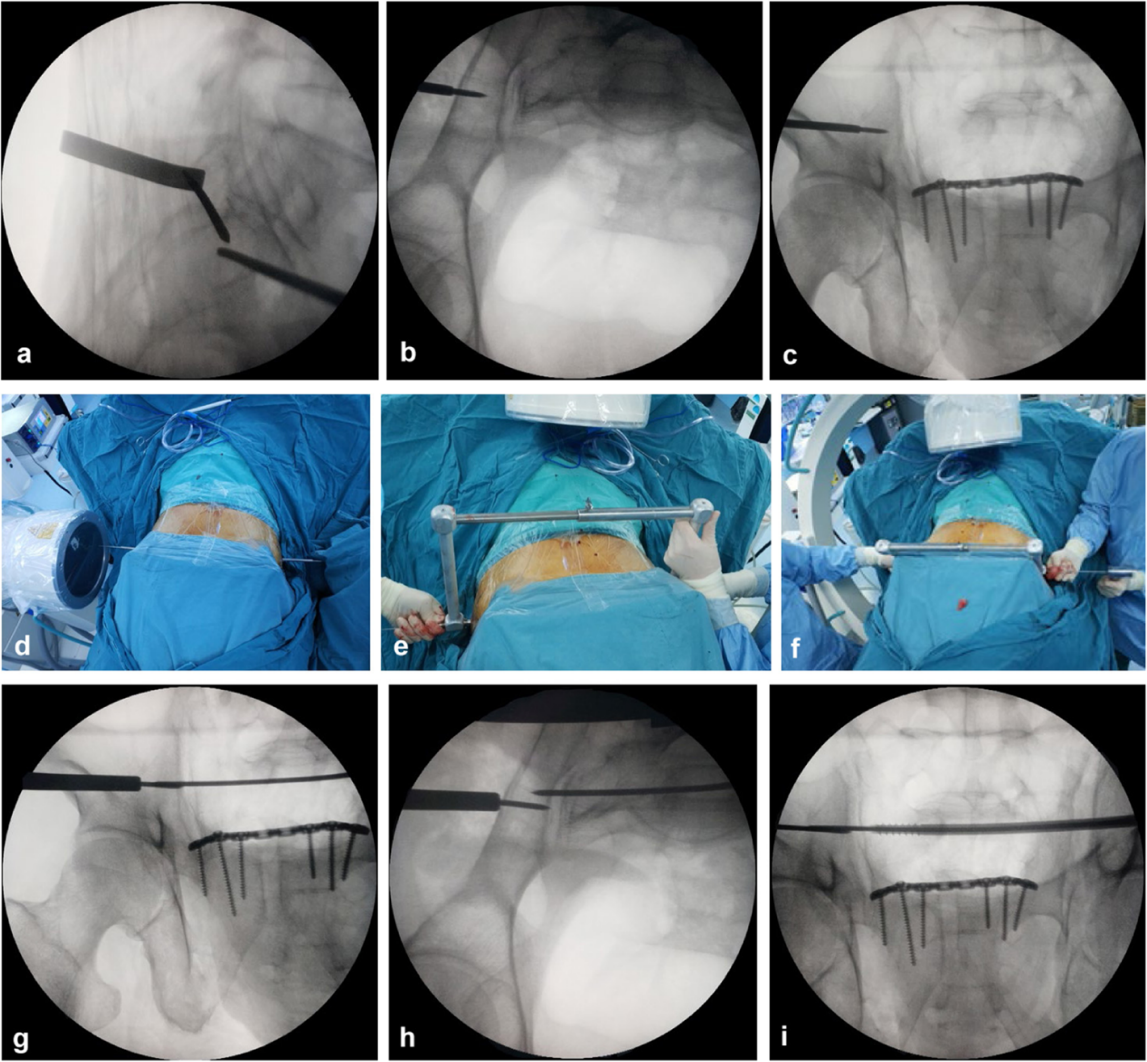
Intraoperative fluoroscopic lateral (**a**), inlet (**b**), and outlet (**c**) views demonstrating the position of the contralateral guidewire was satisfactory. The first cannula guided the starting point of the guidewire and the other cannula guided the exiting point (**d**). The point-to-point coaxial guide apparatus was installed and locked (**e**). A longer guidewire was inserted into the screw corridor along the trajectory directed by the cannulas (**f**). The position of the longer guidewire can be verified by progressive fluoroscopic evaluation (**g**, **h**). When the guidewire exited the contralateral iliac cortex, we inserted into the transiliac-transsacral screw over the guidewire (**i**)

First, a true lateral sacral view is the ideal starting image for the procedure to determine the safe zone and starting point of the guidewire of transiliac-transsacral screw (Fig. 2d). We inserted a threaded guide wire with 2.5-mm diameter into the starting point through the skin onto the lateral ilium on the fracture side (Fig. 2e). The inlet and outlet fluoroscopic views are used to confirm its position. Then, we inserted the cannula along the guide wire, contacted with the ilium cortex, and its serrated tip was inserted into the cortex (Fig. 2f, g). If the guidewire strayed off course, we used another guidewire for slight corrections. It deserves to be mentioned in this first step; we only pay attention to ensure the starting point of the cannula in the safety zone. Then, the other guidewire and cannula of the contralateral side were placed at the exiting point within the safe zone using the same method (Fig. 3a–c). At this time, the first cannula guided the starting point of the guidewire and the other cannula guided the exiting point (Fig. 3d).

**Fig. 4:**
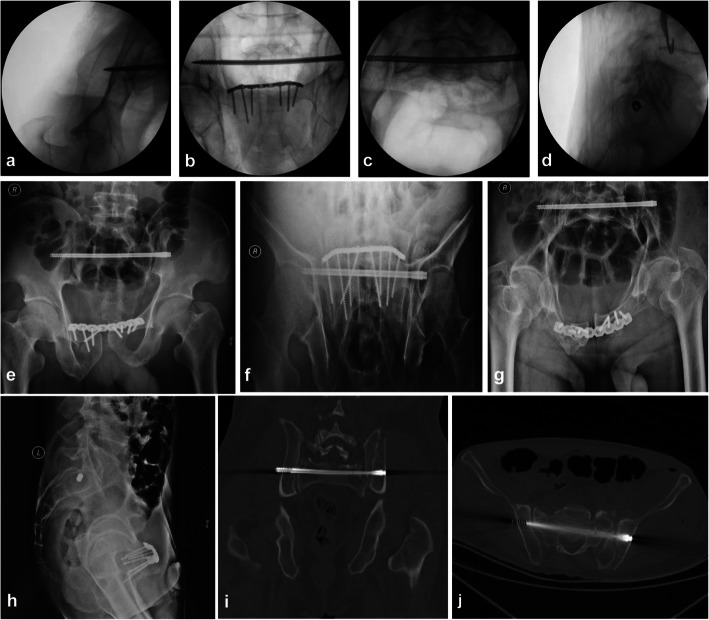
Ensuring the position of the screw by the inlet, outlet and lateral views again, and taking an over-the-top view to assess screw length and far cortex penetration of the iliac (**a**–**d**). As shown in the radiographs and CT scans, the position of the transiliac-transsacral screw was satisfied except for the threads protruding through the contralateral ilium (**e**–**j**)

Next, the point-to-point coaxial guide apparatus was installed and fixedly locked (Fig. 3e). The two cannulas formed a point-to-point coaxial relationship by the guide apparatus. Since the trajectory was directed by the two cannulas from the starting point of the safety zone to the exiting point, they formed a safe point-to-point coaxial straight transiliac-transsacral screw corridor. Subsequently, the first guidewire on the fracture side was removed and a longer guidewire was inserted along the trajectory directed by the cannulas (Fig. 3f). The longer guidewire insertion requires sequential and progressive fluoroscopic evaluation until it is just short of exiting the contralateral iliac cortex (Fig. 3g–i). Then, we removed the guide apparatus and inserted into an appropriate length transiliac-transsacral screw over the guidewire. Ultimately, we ensured the position of the screw by the inlet, outlet, and lateral views again, and took an anteroposterior (AP) rollover view to assess screw length and far cortex penetration of the iliac (Fig. 4a–d). Postoperative radiographs and CT scans are obtained to verify reduction and implant placement (Fig. 4e–j).

#### Free-hand group

The operation procedure was the same as traditional transiliac-transsacral screw insertion under conventional fluoroscopy with free-hand [2].

### Statistical analysis

The data were analyzed using SPSS 20.0 software (Chicago, IL, USA). The independent two-sample *t* test was used for continuous data (presented as mean ± SD) including age, follow-up time, operation time, and radiation exposure times. The chi-square test and Fisher’s exact test was used for discrete data between two groups, including gender, age distribution, injury causes, OTA/AO classification, and screws level. The Wilcoxon rank sum test was used for the data of function outcomes and quality of reduction. *P* value less than 0.05 was considered as statistically significant.

## Results

From July 2017 to December 2019, a total of 38 patients diagnosed with unstable posterior pelvic ring injuries were included in this study. There were no significant differences between the two groups in gender, age, injury causes, pelvic fracture type, and screws level (*P* > 0.05) (Table 1). From the date of surgery, all patients were required to visit our specialist clinic at 1, 2, 3, 6, 12, and 18 months postoperatively. Some patients may lose to follow-up because of multiple reasons. However, no significant differences were found between the two groups (14.7 ± 2.2 months of follow-up in the guide apparatus group vs 13.8 ± 2.1 months in the free-hand group, *P* > 0.05) (Table 1). All screws were not routinely removed.

**Table 1:** Statistic analysis of clinical indicators of the two groups

Variable	PTP (*n* = 20)	Free-hand (*n* = 18)	T/*χ*^2^	*P* value
Age (years)	47.2 ± 17.0	44.7 ± 13.4	0.483	0.632
Gender (women/men)	6/14	7/11	0.333	0.734
Follow-up in months	14.7 ± 2.2	13.8 ± 2.1	1.187	0.243
Cause of injury				
Fall from height/ traffic accident	3/17	4/14	0.329	0.687
Pelvic fracture type (OTA/AO classification)				
Type B/ type C	9/11	12/6	1.799	0.210
Screws level (6.5 mm)				
S1 level/S2 level	9/11	11/7	0.986	0.352
Intraoperative data (mean ± SD)				
Operation time (min)	25.8 ± 4.7	40.5 ± 5.1	− 9.160	< 0.001^a^
Radiation exposure times (N)	24.4 ± 6.0	51.6 ± 8.4	− 11.489	< 0.001^a^

*PTP* point to point guide apparatus

^a^Statistical significance

The patients with anterior pelvic ring fractures were treated with reconstruction plates, anterior column screws, or external fixation devices. The average operative time of the point-to-point guide apparatus group for each screw was significantly less than that in the free-hand group (25.8 ± 4.7 min vs 40.5 ± 5.1, *P* < 0.001). The radiation exposure times were significantly lower in the point-to-point guide apparatus group than that in the free-hand group (24.4 ± 6.0 vs 51.6 ± 8.4, *P* < 0.001) (Table 2).

**Table 2:** Postoperative clinical indicators of the two groups

Variable	PTP (*n* = 20)	Free-hand (*n* = 18)	Range or percent
Function outcomes (Majeed scores, *N*)			*χ*2 = − 0.711, *P* = 0.477
Excellent/good	12/7	9/7	95% vs 88.9%
Fair	1	2	5% vs 11.1%
Quality of reduction (Matta radiology criteria, *N*)			*χ*2 = − 0.107, *P* = 0.914
Excellent/good	14/4	12/5	90% vs 94%
Fair	2	1	10% vs 6%
Screw position			
Intraosseous	16	9	80% vs 50%
Juxtacortical	4	8	20% vs 44.4%
Extraosseous	0	1	0% vs 5.6%
Complications	No	No	

*PTP* point-to-point guide apparatus, *N* number, *vs* versus

A total of 38 transiliac-transsacral screws (6.5 mm cannulated screw) were placed in 38 patients: 20 screws (9 S1, 11 S2) in point to point guide apparatus group and 18 screws (11 S1, 7 S2) in free-hand group. The intraosseous and juxtacortical rate of screw placement in point-to-point guide apparatus group was 100% and higher than 94.4% in the free-hand group. No complications including nerve palsy and revisions were noted postoperatively. According to Majeed scores at 6 months and Matta radiology criteria, function outcomes and quality of reduction has no significant differences between the two groups. (*χ*^2^ = − 0.711, *P* = 0.477; *χ*^2^ = − 0.107, *P* = 0.914) (Table 2).

## Discussion

Several studies have described the relationship of the iliac cortical density (ICD), that is, the dense area of anterior ilium located directly lateral to the sacroiliac joint, and the sacral alar anterior cortical anatomy. This relationship is paramount for identifying the safe zone and an appropriate starting point [16]. On the true lateral sacral fluoroscopic image, the ICD is coplanar with the anterior sacral alar cortical bone in the nondysmorphic sacrum. But in patients with sacral dysmorphism, like a more acute alar slope, would have less alar bone available for screw insertion [17]. This oblique dysmorphic alar osteology makes transiliac-transsacral screw fixation impossible [12, 16]. However, patients with a dysmorphic sacrum always have a safe zone at the second sacral segment that can insert a transiliac-transsacral screw [11].

The so-called safe zone, most restricted between the sacral ala and sacral neural tunnel, is the area within the sacrum where completely intraosseous transiliac-transsacral screws are ideally inserted [16, 18]. Since the pelvis can be considered as a structure of bilateral symmetry and there are two safe zones for transiliac-transsacral screw at both sides of ilium [19], we regard the transiliac-transsacral screw corridor as a point-to-point (safe zone to safe zone) coaxial trajectory from the starting point of the guidewire to the exiting point. The two points are included in the safe zone of the transiliac-transsacral screw estimated by a true sacral lateral view. On the true lateral view, two points ideally will overlap to form one point. Since two points form a coaxial straight line, the exiting point, representing the starting point at the contralateral side, can be used to find and guide the trajectory and direction of the guidewire of the transiliac-transsacral screw. This is the core principle and the most important safety aspect of the point-to-point coaxial guide apparatus.

It has been reported that 3D fluoroscopic navigation enabled safer iliosacral screw placement [20]. However, Takao et al. found that the risk factors for inaccurate positioning of iliosacral screws inserted using 3D fluoroscopic navigation were the screw insertion angle in the axial plane and the use of transsacral screws because the navigation system guides the sleeve device for guidewire insertion, not the guidewire or the screw itself; and the guidewire flexibility is also one possible reason [21]. In our study, the screw insertion angle was not a risk factor for inaccurate screw insertion as the transsacral trajectory was guided by the guide apparatus according the principle mentioned above. Moreover, although the guidewire has flexibility, the corridor of the guidewire formed by the guide apparatus is not affected by the elasticity of the skin and soft tissue around the cannulas, even in patients with truncal obesity, which allow the surgeon to keep the direction of the guidewire during inserting.

Medical robotic systems for percutaneous iliosacral screw placement have been developed [22]. Wang et al. reported that the accuracy of the robot-assisted technique was superior to the conventional free-hand technique [23]. But, using a robot-assisted navigation system adds to the cost of the surgery and many hospitals may have difficulty paying for robots [23], and there were no reports about the accuracy of the robot-assisted technique for transiliac-transsacral screw placement in the treatment of posterior ring unstable pelvic fracture.

In our study, accuracy of the point-to-point coaxial guide apparatus was superior to that of the free-hand technique. Our clinical experience with the described screw placement technique resulted in the intraosseous and juxtacortical rate of screw placement (100%) higher than in the free-hand group (94.4%). Also, the time required to place each screw and the radiation exposure times in the guide apparatus group was significantly better than in the free-hand group, and no complications were noted at the final follow-up. Although much time was spent in finding the exiting point, the duration of the operation and the number of fluoroscopy exposures were significantly reduced, and there are no expensive surgical instruments required because the manufacture of the guide apparatus is simpler and cheaper than a robot, CT machine, or 3D navigation [24]. Ultimately, surgeons who are adept at inserting iliosacral and transiliac-transsacral screws can master this technique with a small learning curve.

However, some limitations are inherent in this study. First, although the learning curve of this technique is small, it still requires surgeons to have rich surgical experience and correctly interpret intraoperative imaging to verify reduction and implant placement. Meanwhile, the fracture or dislocation of the sacroiliac joint may make the standard lateral view difficult to achieve; this could make it hard for us to find the exact starting point and exiting point of the guide wire. So, an accurate sacroiliac joint reduction is extremely important for this technique and it must already be achieved. Finally, this was a retrospective evaluation of our experience; a prospective study and a more sufficient sample size might give further insights in the limitations and complications of the guide apparatus.

## Conclusion

The point-to-point coaxial guide apparatus is feasible for assisting the transiliac-transsacral screw in the treatment of posterior unstable pelvic fractures. Accuracy of the point-to-point coaxial guide apparatus was superior to that of the free-hand technique. It has the advantages of simple operation, reasonable design, and no need for expensive equipment, and provides an additional surgical strategy for the insertion of the transiliac-transsacral screw.

## Data Availability

All data analyzed during this study are included in this published article and its supplements.
